# D-Chiro-Inositol and Myo-Inositol Induce WAT/BAT Trans-Differentiation in Two Different Human Adipocyte Models (SGBS and LiSa-2)

**DOI:** 10.3390/ijms24087421

**Published:** 2023-04-18

**Authors:** Giovanni Monastra, Riccardo Gambioli, Vittorio Unfer, Gianpiero Forte, Elsa Maymo-Masip, Raffaella Comitato

**Affiliations:** 1 Systems Biology Group Lab, 00161 Rome, Italy; 2 Experts Group on Inositols in Basic and Clinical Research (EGOI), 00161 Rome, Italy; 3 R&D Department, Lo.Li. Pharma, 00156 Rome, Italy; 4 UniCamillus-Saint Camillus International University of Health Sciences, 00131 Rome, Italy; 5 Institut Investigació Sanitària Pere Virgili (IISPV), 43003 Tarragona, Spain; 6 CIBER de Diabetes y Enfermedades Metaboílicas Asociadas (CIBERDEM)-Instituto de Salud Carlos III, 28029 Madrid, Spain; 7 Council for Agricultural Research and Economics—Research Centre for Food and Nutrition, 00178 Rome, Italy

**Keywords:** brown adipose tissue, white adipose tissue, D-chiro-inositol, myo-inositol, obesity, trans-differentiation, UCP-1, PPAR-γ, estrogen receptor

## Abstract

White adipose tissue/brown adipose tissue trans-differentiation is one of the main study targets for therapies against obesity and metabolic diseases. In recent years, numerous molecules able to induce such trans-differentiation have been identified; however, their effect in obesity therapies has not been as expected. In the present study, we investigated whether myo-inositol and its stereoisomer D-chiro-inositol could be involved in the browning of white adipose tissue. Our preliminary results clearly indicate that both, at 60 μM concentration, induce the upregulation of uncoupling protein 1 mRNA expression, the main brown adipose tissue marker, and increase mitochondrial copy number as well as oxygen consumption ratio. These changes demonstrate an activation of cell metabolism. Therefore, our results show that human differentiated adipocytes (SGBS and LiSa-2), assume the features typical of brown adipose tissue after both treatments. Furthermore, in the cell lines examined, we proved that D-chiro-inositol and myo-Inositol induce an increase in the expression of estrogen receptor mRNAs, suggesting a possible modulation by these isomers. We also found an increase in the mRNA of peroxisome proliferator-activated receptor gamma, a very important target in lipid metabolism and metabolic diseases. Our results open new opportunities for the use of inositols in therapeutic strategies to counteract obesity and its metabolic complications.

## 1. Introduction

A pivotal study carried out in 195 countries over 25 years by the collaborators of the Global Burden of Disease [1] revealed that, in 2015, 107.7 million (5%) children and 603.7 million (12%) adults were obese, worldwide [2]. Obesity is a chronic metabolic disease caused by different factors: genetic, environmental, psychological, and social. It is characterized by long-term imbalance between energy intake and energy expenditure and by an imbalance of white adipose tissue (WAT) and brown adipose tissue (BAT). 

There are three distinct adipocyte types in humans, namely, white, brown, and beige adipose tissue, according to their function and morphology [3,4,5,6]. Essentially, WAT and BAT play antagonistic functions; WAT works as fat storage and energy stock, while BAT dissipates energy producing heat, also helping to maintain internal body temperature. White adipocytes have a size ranging from 20 to 200 μm, contain few mitochondria and only one vacuole that occupies ~90% of cell volume, where lipids are stored and mobilized during the energy expenditure phase. These cells from WAT give rise to several fat depots that are distributed in two anatomical compartments of the body: subcutaneous and visceral. In contrast, brown adipocytes have a large number of mitochondria, containing a unique protein known as uncoupling protein 1 (UCP-1). The high number of mitochondria is also responsible for the characteristic staining of this tissue. Furthermore, BAT cells are characterized by multilocular lipid droplets and are smaller (15–50 μm) than WAT adipocytes. BAT tissue does not store energy but dissipates it through thermogenesis.

In the past three decades, fat cells similar to brown adipocytes were also identified. They are beige in color and positive for the expression of UCP-1, that “appears” in response to specific stimuli such as physical exercise, exposure to cold or some hormones. Unlike BAT, they can accumulate in typical WAT deposits and were named beige or “brite” adipocytes (a combination of the English terms brown and white). Although beige adipocytes display characteristics similar to BAT cells, they have different anatomical locations. In fact, the beige adipocytes are detectable in the subcutaneous regions where normally WAT occurs, and BAT is absent [7].

Therefore, based on what is currently known and currently used drugs, the future strategies to fight obesity could involve not only diets, fat absorption and inhibition or decrease in appetite, but also treatments with drugs. Current drugs used to treat obesity include: (1) orlistat, an inhibitor of gastrointestinal lipase that decreases fat absorption; (2) the association between phentermine and topiramate, an amphetamine-like compound and an anti-convulsing drug that seems to inhibit the appetite; (3) liraglutide, an agonist of the receptor of glucagon-like peptide 1, which induces a delay in gastric emptying and decreases appetite [8]. Moreover, treatments with specific drugs that induce WAT/BAT trans-differentiation could be particularly useful to treat obesity. So far, only a few drugs have been approved in the world for obesity therapies; however, their effect has not been as expected. Therefore, it is necessary to find novel compounds that can induce WAT/BAT trans-differentiation and evaluate their efficacy in human obesity therapies. In our study, we tested the effects of two natural molecules involved in several metabolic pathways, the stereoisomers myo-inositol (myo-Ins), which constitutes over 99% of the inositol pool, and D-chiro-inositol (D-chiro-Ins). An insulin-dependent epimerase gives rise to D-chiro-Ins from myo-Ins, especially in the liver, muscles, and blood, where the highest conversion rate (~60%) is found [9]. Both myo-Ins and D-chiro-Ins, in the form of inositol phosphoglycan, are insulin second messengers and influence cell metabolism, by activating key enzymes involved in oxidative and non-oxidative glucose metabolism. One of the most significant activities of both myo-Ins and D-chiro-Ins concerns glycemia regulation. Specifically, D-chiro-Ins participates in glycogen synthesis, while myo-Ins promotes glucose cellular uptake [10,11]. As therapeutic agents, both stereoisomers exert an insulin-mimetic activity and are also effective against insulin resistance [10,11]. They also play other pivotal physiological roles, such as promoting ovulation and fertility [12,13]. Moreover, other studies reported that dietary myo-Ins deficiency could result in lipid accumulation in the liver of rats [14] and fish [15,16]. D-chiro-Ins also plays a key role in lipid metabolism regulating steroid production in rats [17] and adipocyte differentiation in human cells [18]. A recent paper highlighted that this stereoisomer, as myo-Ins, can upregulate the gene expression of peroxisome proliferator-activated receptor gamma (PPAR-γ) [17].

In recent years, myo-Ins and D-chiro-Ins were widely used in several treatments, the most important in patients with polycystic ovary syndrome, due to the physiological and therapeutic properties that emerged, and are still emerging, from many experimental and clinical studies. Polycystic ovary syndrome is a complex condition that is diagnosed from the presence of two out of three criteria: anovulation; hyperandrogenism; and cystic ovaries. These patients are usually affected by insulin resistance, and clinical evidence exists on the use of myo-Ins and D-chiro-Ins in these patients.

We tested these compounds in two different human adipocyte models, Simpson–Golabi–Behmel syndrome cells (SGBS) and LiSa-2, subcutaneous and visceral adipocytes, respectively. Indeed, both the cell lines consist of adipocyte-like cells, and such a tendency to differentiate into adipocyte makes them the perfect models to mimic an in vivo condition and study the effect on the differentiation and the browning processes of a supplementation with inositols [19]. The results described here showed that in such models both myo-Ins and D-chiro-Ins could induce WAT/BAT trans-differentiation, probably activating the estrogen signaling pathway and/or modulating PPAR-γ gene expression.

## 2. Results

### 2.1. Modulation of UCP-1 mRNA

**Figure 1:**
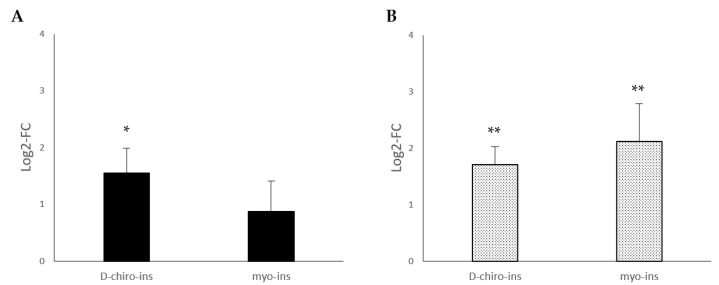
Modulation of the UCP-1-messenger expression in two adipocyte cell lines, (**A**) SGBS cells, subcutaneous fat and (**B**) LiSa-2 cells, visceral fat. D-chiro-Ins and myo-Ins concentrations used for the experiments were 60 μM. Results are reported as mean ± SD of data obtained from 3 independent experiments in technical triplicate. Bars show log 2-fold change (treated vs. control). Data were analyzed by one-way ANOVA. *p*-values ≤ 0.05 were considered statistically significant. * *p* < 0.05 versus control; ** *p* < 0.01 versus control.

Figure 1 reports the modulation of UCP-1 mRNA, the main marker of the trans-differentiation process, in SGBS and LiSa-2 cells, after treatment with D-chiro-Ins (60 μM) and myo-Ins (60 μM). Following treatment with D-chiro-Ins, UCP-1 mRNA was over expressed in both SGBS and LiSa-2 cells, whereas myo-Ins induced a significant positive modulation only in visceral adipose tissue cells (LiSa-2). Our data suggest that D-chiro-Ins and myo-Ins can induce WAT/BAT trans-differentiation through UCP-1 activation.

### 2.2. Increase in Mitochondrial DNA Copy Number

**Figure 2:**
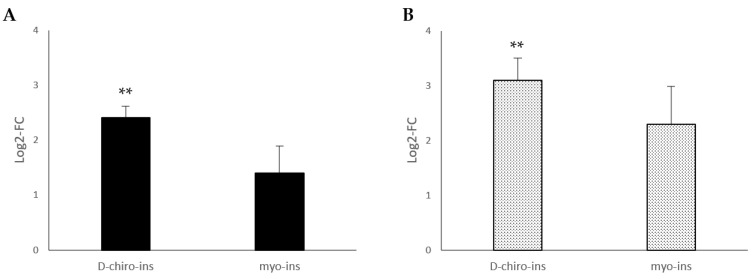
Modulation of mitochondrial DNA copy number in (**A**) SGBS cells and (**B**) LiSa-2 cells. SGBS and LiSa-2 differentiated adipocytes were treated with D-chiro-Ins (60 μM) and myo-Ins (60 μM) for 72 h, with a single medium change at 36 h. Results are reported as mean ± SD of data obtained from 3 independent experiments in technical triplicate. Relative DNA mitochondrial copy number was calculated from the difference between the threshold cycle (CT) values for mitochondrial DNA and nuclear specific amplification. Bars show log 2-fold change (treated vs. control). Data were analyzed by one-way ANOVA. *p*-values ≤ 0.05 were considered statistically significant. ** *p* < 0.01 versus control.

To confirm the ability of D-chiro-Ins and myo-Ins to induce WAT/BAT trans-differentiation, we also evaluated mitochondrial DNA copy number by quantitative real-time polymerase chain reaction (PCR). In fact, metabolically active adipose tissue increases energy dissipation by increasing the numbers of mitochondria. As can be seen in Figure 2, mitochondrial DNA increased both in SGBS and LiSa-2 cells, but a significant increase occurred only following D-chiro-Ins treatment.

### 2.3. Oximetry Assessment

**Figure 3:**
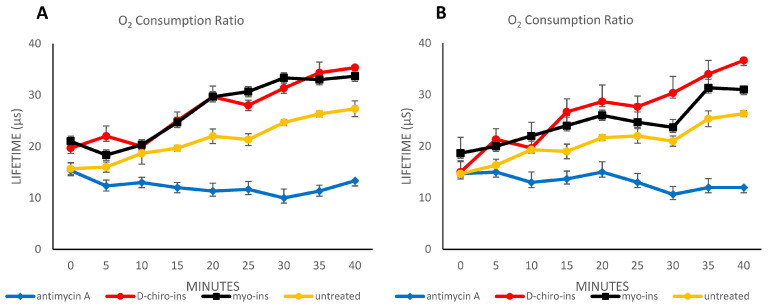
Oxygen consumption ratio in SGBS cells (**A**) and LiSa-2 cells (**B**). SGBS and LiSa-2 differentiated adipocytes were treated with D-chiro-Ins (60 μM) and myo-Ins (60 μM) for 72 h, with a single medium change at 36 h. Results are reported as mean ± SD of data obtained from 3 independent experiments in technical triplicate. Data were analyzed by one-way ANOVA. *p*-values ≤ 0.05 were considered statistically significant.

As a further confirmation of D-chiro-Ins and myo-Ins ability to induce WAT/BAT trans-differentiation, we assessed the oxygen consumption ratio of the differentiated adipocytes (both SGBS and LiSa-2) after 72 h of treatment. Antimycin was used as a negative control. Our experiments clearly show (Figure 3A,B) that an increase in oxygen consumption is found in both SGBS and LiSa-2 cells, indicating a greater functioning of the mitochondria in the two cell lines. We also included the experiments performed on untreated cells. The treatment induced an increase in oxygen consumption with respect to untreated cells. This is particularly evident in all the timepoints for SGBS cells, except for the 10 min timepoint (Figure 3A). In the case of LiSa-2 cells (Figure 3B), the treatment with D-chiro-Ins recorded significant differences in oxygen consumption except for the 10 min timepoint, while myo-Ins-treated cells exhibited higher oxygen consumption with respect to untreated cells in all the timepoints except for 25 min. The only significant differences between the treatments with myo-Ins and D-chiro-Ins were recorded at 5 and 25 min in SGBS cells and at 30 and 40 min in LiSa-2 cells.

### 2.4. Expression of Estrogen Receptors (ERα and ERβ) mRNAs

**Figure 4:**
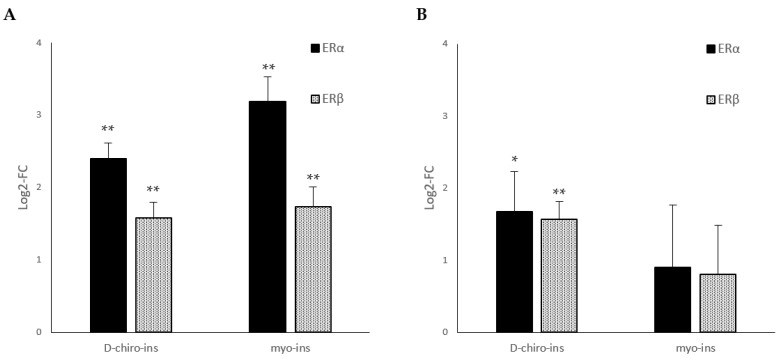
Modulation of estrogen receptor mRNA expression in (**A**) SGBS and (**B**) LiSa-2 differentiated adipocytes treated with D-chiro-Ins (60 μM) or myo-Ins (60 μM) for 72 h, with a single medium change at 36 h. Results are reported as mean ± SD of data obtained from 3 independent experiments in technical triplicate. Bars show log 2-fold change (treated vs. control). Data were analyzed by one-way ANOVA. *p*-values ≤ 0.05 were considered statistically significant. * *p* < 0.05; ** *p* < 0.01 versus control.

We observed (Figure 4) that D-chiro-Ins and myo-Ins increase estrogen receptor α (ERα) and estrogen receptor β (ERβ) mRNA levels in both cell lines. In particular, we noticed by optical microscopy that the upregulation of ERα and ERβ mRNAs in SGBS cells (Figure 4A) was associated with the formation of many lipid droplets with respect to untreated cells (data not shown), a typical feature shown by differentiated SGBS cells. On the other hand, we also observed a significant increase in ERα and ERβ mRNAs in LiSa-2 cell lines only following D-chiro-Ins treatment (Figure 4). Therefore, we supposed that D-chiro-Ins and myo-Ins could induce WAT/BAT trans-differentiation mediated by classical estrogen-triggered pathways.

### 2.5. Increase in PPAR-γ Gene Expression

**Figure 5:**
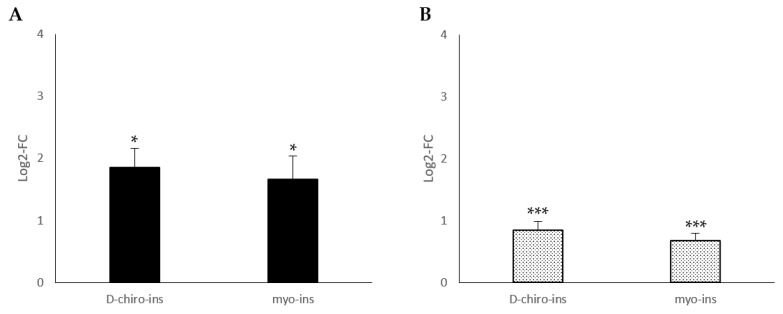
Modulation of the expression for the PPAR-γ variant 1 (PPAR-γ v1) messenger in (**A**) SGBS and (**B**) LiSa-2 adipocyte cells. D-chiro-Ins and myo-Ins concentrations used for the experiments were 60 μM. Results are reported as mean ± SD of data obtained from 3 independent experiments in technical triplicate. Bars show log 2-fold change (treated vs. control). Data were analyzed by one-way ANOVA with repeated measures followed Duncan–Waller post hoc test. *p*-values ≤ 0.05 were considered statistically significant. * *p* < 0.05 versus control; *** *p* < 0.001 versus control.

**Figure 6:**
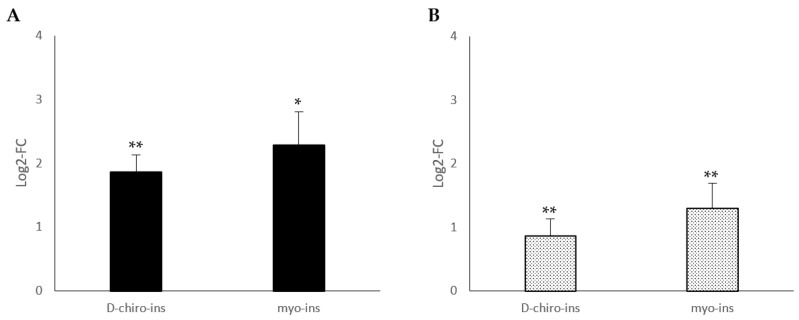
Modulation of the expression for the PPAR-γ variant 2 (PPAR-γ v2) messenger in (**A**) SGBS and (**B**) LiSa-2 adipocyte cells. D-chiro-Ins and myo-Ins concentrations used for the experiments were 60 μM. Results are reported as mean ± SD of data obtained from 3 independent experiments in technical triplicate. Bars show log 2-fold change (treated vs. control). Data were analyzed by one-way ANOVA with repeated measures followed Duncan–Waller post hoc test. *p*-values ≤ 0.05 were considered statistically significant. * *p* < 0.05 versus control; ** *p* < 0.01 versus control.

Finally, we also tested the mRNA expression of the two variants of PPAR-γ (variant 1 and variant 2), given their already identified role in WAT/BAT trans-differentiation and in lipid metabolism. As shown in Figure 5 and Figure 6, the two variants were significantly upregulated in SGBS and in LiSa-2 cells for both treatments with inositol stereoisomers. In our experiment, we did not observe any significant difference for PPAR-γ variants between D-chiro-Ins and myo-Ins treatments.

## 3. Discussion

In our experiments, we demonstrated that D-chiro-Ins and myo-Ins induce WAT/BAT trans-differentiation in the two principal adipocyte human cell models: SGBS and LiSa-2. SGBS cells are non-immortalized pre-adipocytes isolated by subcutaneous fat tissue, whereas LiSa-2 is a liposarcoma of visceral fat. Both cell models are able to trans-differentiate with specific external stimuli.

A fundamental aspect of BAT/beige regulation is the stimulation of UCP-1 [20]. UCP-1 is expressed only in brown/beige adipocytes. It increases the proton conductance in the inner mitochondrial membrane of these specific adipocytes, thus determining the uncoupling of the respiratory chain and the production of heat. This action of UCP-1 in BAT is the main molecular basis for thermogenesis in homeothermic mammals in response to exposure to cold and diet [21]. In our experiments, D-chiro-Ins treatment induced an increase in UCP-1 mRNA levels in both cell lines (visceral and subcutaneous adipose tissue), while for myo-Ins, a significant positive modulation was detected only in visceral adipose tissue cells (LiSa-2), showing a differentiation of adipocyte towards BAT-type characteristics. These results were confirmed by the increase in mitochondrial DNA copy number, even if such an effect was only observed in the D-chiro-Ins treatments, in both adipocyte cell models. Finally, an increased consumption of extracellular oxygen was found in SGBS and in LiSa-2 adipocytes, for both inositol stereoisomer. Our data suggest that D-chiro-Ins could promote WAT/BAT trans-differentiation, regardless of the cell lines used, while myo-Ins seems to act mainly on visceral fat. Despite the non-significance of myo-Ins treatment in increasing mitochondrial DNA, the increase in oxygen consumption likely suggests that myo-Ins can induce trans-differentiation even in subcutaneous fat. Moreover, as the cells can convert myo-Ins to D-chiro-Ins when necessary, we may hypothesize that this happened in our model. Therefore, even though a significant change in UCP-1 was not detected following myo-Ins treatment, the synergistic action of myo-Ins and de novo synthesized D-chiro-Ins may explain the observed downstream effects, such as the increased oxygen consumption. Such an increase in oxygen consumption and in thermogenesis determines an increase in energy expenditure, which counterbalances the energy intake, thus helping to maintain a stable body weight. Growing evidence supports the idea that BAT and beige tissue-mediated thermogenesis contributes to energy dissipation, preventing and/or counteracting obesity. This hypothesis is strengthened by in vivo studies suggesting that the activation of BAT and beige thermogenesis induces weight loss and WAT reduction in mice [22,23]; likewise, an observational study of obese subjects highlighted the pivotal role of beige tissue in the maintenance and loss of weight [24].

We have observed that, in SGBS and LiSa-2 cells, treatment with D-chiro-Ins and myo-Ins induced an upregulation of ER (ERα and ERβ) mRNA. Our results confirm previous experimental data regarding the role of the estrogen signaling pathway in adipose tissue and in obesity disease. In fact, recent reports show that estradiol stimulates BAT metabolism and induces WAT beiging [25,26], and the expression of ESR1, the gene encoding ERα, is reduced in WAT from obese women [27]. Zhou and coworkers [28] have demonstrated that the expression of ESR1 (ERα) is inversely associated with fat mass and regulates mitochondrial function and energy homeostasis in WAT and BAT via the coordinated control of mitochondrial DNA replication. These data suggest that a similar mechanism occurred also in our experimental model. Moreover, adipose tissue is the most important site for estrogen production outside the gonads due to the presence of the enzyme aromatase, belonging to the family of cytochrome P450, which converts androgens taken up from the circulation into estrogens [29]. Due to the presence of aromatase in adipose tissue, the locally produced estrogen can affect metabolism regardless of plasma estradiol levels [29]. Estrogen signaling is among the most significant regulator of BAT activity and differentiation [30]. As was indirectly confirmed, the thermogenic activity and UCP-1 mRNA expression in BAT are reduced by ovariectomy [31,32], while administration of estradiol to ovariectomized mice induces UCP-1 expression in BAT [33]. Other experiments carried out in primary cultures of murine brown adipocytes confirmed that the estrogen pathway has a direct activating effect on brown adipocytes; for instance, by inducing the mitochondrial biogenesis factors [29]. The mechanism by which estrogens promote brown adipocyte proliferation and differentiation, including UCP-1 mRNA expression, is likely driven by ERα [34]. Considering our observations, we hypothesized that D-chiro-Ins and myo-Ins could perform their WAT/BAT trans-differentiation effects by an undefined mechanism involving the estrogen receptor; these results are also supported by a recent study by Montt-Guevara and collaborators [18], who demonstrated that D-Chiro-Ins can regulate human adipocytes (SGBS) directly, by enhancing their differentiation and insulin receptor signaling in synergy with estrogen. Nonetheless, two clinical trials in men showed that treatment with D-chiro-Ins significantly increased androgen levels and decreased estrogen levels [35,36], supporting its current therapeutic use in specific pathologies, i.e., those characterized by excessive estrogens or reduced androgens [37,38,39]. In this scenario, our current data opens a new window on the effects of these compounds and indicates that the mechanism of action of D-chiro-Ins and myo-Ins is more complex than previously thought. Therefore, further studies will be needed to investigate the role of ERs in WAT/BAT trans-differentiation processes.

Finally, we also assessed the expression of PPAR-γ, a very important target in lipid metabolism and metabolic diseases [40,41], that is induced by myo-Ins [42] and by D-chiro-Ins [17]. PPAR-γ is highly expressed in both brown and white adipocytes and its activation induces the trans-differentiation of brown adipocytes [43]. Our data confirm the role of myo-Ins and D-chiro-Ins in the upregulation of PPAR-γ. Moreover, we have demonstrated, for the first time, that myo-Ins and D-chiro-Ins can modulate the expression of two variants of PPAR-γ, v1 and v2. The two variants are the product of an alternative splicing of the same gene [44]. In particular, the PPAR-γ1 isoform lacks the first 30 N-terminal amino acids compared to PPAR-γ2. In the presence of PPAR-γ ligands, both variants can induce adipogenesis, but PPAR-γ2 responds more efficiently to low ligand concentrations [45]. Despite functional similarities [46], the PPAR-γ1 isoform is ubiquitously expressed, while PPAR-γ2 is limited to adipose tissue [47]. The finding that both myo-Ins and D-chiro-Ins stimulate PPAR-γ2 clearly indicates that both the isomers play a role in adipose tissue. Moreover, as inositols are commonly detectable in cellular membranes, we can speculate that the regulation herein reported originates from the physiological mechanisms of browning. Indeed, our work strongly suggests that inositols could represent valid molecules to treat obesity, as they could promote browning of both subcutaneous and visceral adipocytes, contributing to weight-loss interventions.

## 4. Materials and Methods

### 4.1. Cell Culture SGBS

The SGBS human preadipocyte line was derived from the stromal vascular fraction of subcutaneous adipose tissue of a male infant with Simpson–Golabi–Behmel syndrome, first described in the early 2000s [48,49]. This syndrome is an X-linked recessive pathology that is considered a congenital disorder and leads to the overgrowth of body districts, including head, kidney, hearth, and tongue. These cells are considered as a really suitable model for studies on the browning and differentiation of adipocytes [49]. These cells, first isolated by professor Wabitsch, were generously sent by him in the proliferative phase (28th generation). SGBS cells are neither transformed nor immortalized and provide an almost unlimited source of cells, due to their ability to proliferate for up to 50 generations without losing their characteristics and their ability to differentiate into mature adipocytes. They provide an excellent experimental model for studying the subcutaneous fat and for investigating mechanisms leading to the browning of WAT.

During the experiments, SGBS cells were amplified not further than the 35th generation in DMEM-F12 medium (Dulbecco’s Modified Eagle’s Medium/Nutrient F-12 Ham) (Sigma-Aldrich, Saint Louis, MO, USA) containing 33 μM biotin (Sigma-Aldrich, Saint Louis, MO, USA), 17 μM pantothenic acid (Sigma-Aldrich, Saint Louis, MO, USA), 100 U/mL penicillin/streptomycin (Sigma-Aldrich, Saint Louis, MO, USA) and 10% FBS (Gibco, Billings, MT, USA). Growth medium was changed every 2 days and cells were cultured at 37 °C in a 5% CO_2_ incubator. Cells were cultured in this medium until complete confluence was reached.

#### Adipocyte Differentiation Process

Differentiation started with SGBS cells at confluence (day 0) in the medium containing 10% FBS. Preadipocytes were washed with PBS, and then changed to the primary differentiation serum-free medium (differentiation A media), for 4 days, containing: biotin 33 μM, pantothenic acid 17 μM, penicillin/streptomycin 100 U/mL, rosiglitazone 2 μM (Cayman Chemicals, Ann Arbor, MI, USA), human apotransferrin 10 μg/mL (Sigma-Aldrich, Saint Louis, MO, USA), human insulin 20 nM (Sigma-Aldrich, Saint Louis, MO, USA), dexamethasone 25 nM (Sigma-Aldrich, Saint Louis, MO, USA), 3-isobutyl-1-methylxanthines 500 μM (Sigma-Aldrich, Saint Louis, MO, USA), cortisol 100 nM (Sigma-Aldrich, Saint Louis, MO, USA) and triiodothyronine 200 pM (Sigma-Aldrich). After the fourth day, the medium was changed. Rosiglitazone, 3-isobutyl-1-methylxanthines and dexamethasone were removed during the remaining 10 days of differentiation (differentiation B media). B media was replaced every four days. Cells were considered fully mature 28 days post-differentiation when clearly visible lipid droplets formed.

### 4.2. Cell Culture LiSa-2

The LiSa-2 human liposarcoma cell line, obtained from a retroperitoneal metastasis of a liposarcoma, was generously sent by professor Wabitsch [19]. LiSa-2 cells are derived from liposarcoma that grow cancerous in the presence of serum, while they retain a differentiated adipocyte-like behavior in the absence of growth factors; thus, they are extensively used in experimental models of visceral fat. LiSa-2 cells were cultured in medium containing serum (DMEM/Ham’s F12 (1:1), 10% FCS, antibiotics), while a serum-free basal medium was used for differentiation (DMEM/F12 (1:1) supplemented with 10 μg/mL transferrin, 15 mM NaHCO_3_, 15 mM HEPES, 33 mM biotin, 17 mM pantothenate, 100 U/mL penicillin and 0.1 mg/mL streptomycin, 1 nM insulin, 20 pM triiodothyronine and cortisol 1 μM). Differentiation into fat cells was evaluated by conventional microscopy (Diavert, Leitz, Germany) at a magnification of 200 times. LiSa-2 cells reached a full differentiation when their cytoplasm was completely filled with lipid droplets [19].

### 4.3. Treatments

Both differentiated cell lines were treated with 60 μM D-chiro-Ins or myo-Ins for 72 h, with a single medium change at 36 h. The selected concentration of inositols was intended to reproduce the concentrations detectable in vivo following inositol supplementation. The concentration was established on the previous findings from our group on the concentrations of both myo-Ins and D-chiro-Ins in vivo [35,50,51,52]. The time of treatment was determined considering the half-life of UCP-1, which may range from 30 to 72 h [53,54]. Finally, the cells were washed with PBS, collected, and used for subsequent experiments.

SGBS and LiSa-2 cells were also tested to exclude mycoplasma contamination. The absence of mycoplasma was verified by PCR analysis (PCR Mycoplasma Test Kit I/C, PromoKine, PromoCell France).

### 4.4. RNA Isolation, RT-PCR, qPCR

Total RNA was isolated from differentiated adipocytes (LiSa-2 and SGBS) using Trizol (Invitrogen Life Technologies, Waltham, MA, USA), according to the manufacturer’s instructions. RNA concentrations were determined by Nanodrop. A retro-transcriptase enzyme (Takara, Osaka, Japan) was used to generate the cDNA, according to the manufacturer’s instructions. The primer sequences are shown in Table 1. Finally, quantitative real-time PCR (qPCR) experiments were performed in an Applied Biosystem 7500 fast (Applied Biosystems, Waltham, MA, USA) as follows: 10 s at 94 °C, followed by 12 s (40 cycles) at 94 °C, 30 s at 60 °C. All experiments were performed in biological and technical triplicate. Normalized gene expressions were calculated with the ΔΔCt method. Human β-actin was used as an endogenous control.

### 4.5. Mitochondrial and Nuclear DNA Isolation and mitDNA Quantification by qPCR

Mitochondrial DNA was isolated from differentiated SGBS and LiSa-2 adipocytes using the Mitochondrial DNA Isolation Kit (Abcam, UK), according to the manufacturer’s instructions. Nuclear DNA was isolated from differentiated SGBS and LiSa-2 adipocytes by Nuclear DNA Isolation Kit (Abcam), according to the manufacturer’s instructions. qPCR experiments were performed in an Applied Biosystem 7500 fast (Applied Biosystems) as follows: 20 min at 95 °C, and 50 cycles of 15 s at 95 °C, 20 s at 58 °C, 20 s at 72 °C. Relative mitochondrial DNA content was calculated from the difference between the threshold cycle (CT) values for mitochondrial DNA and nuclear specific amplification. During the qPCR, we used diluted samples, 10 μM from each primer (human mitochondrial DNA specific primers: forward 5′-TTCTGGCCACAGCACTTAAA -3′, reverse 5′-TGGTTAGGCTGGTGTTAGGG-3′, nuclear specific primers (SIRT1 gene): forward 5′-GCAGGCATTCCTGGAAGAG-3′, reverse 5′-TGTGTGCCCTACACAATGC-3′).

### 4.6. Ex Vivo Oxygen Consumption Rate Measurement

Oxygen consumption assay was performed using an oxygen consumption assay kit (CAT: ab197243, Abcam, Waltham, MA, USA). Briefly, differentiated SGBS and LiSa-2 adipocyte cell lines (8.0 × 104 cells/well) were plated on 96-well cell-culture plates and incubated overnight. After removing the media from all wells, they were replaced with 150 μL of fresh culture media. Reconstituted extracellular O_2_ consumption reagent (10 μL) was added to each sample well. For the blank control well, 10 μL of fresh culture medium was added. Each well was sealed by adding 100 μL of prewarmed high-sensitivity mineral oil. For the measurement, the prepared plate was inserted into a fluorescence plate reader preset to the measurement temperature (37 °C). All controls and samples were carried out in technical triplicate. The extracellular O_2_ consumption signal at 5 min intervals for 30 min at Ex/Em = 380/650 nm was measured by Tecan Infinite F200 PRO microplate reader. In the assay, as indicated, mitochondrial respiration depleted the oxygen within the assay medium, quenching of the fluorescent dye was reduced, and the fluorescence signal increased proportionally.

Data are expressed as the percentage effect of lifetime (μs; see Equation (1)) for the treatment relative to the untreated control:

Lifetime (μs) (T) = (D2 − D1)/ln (W1/W2)(1)

where W1 and W2 are the times for the dual measurement windows and D1 and D2 are the delay times prior to W1 and W2, respectively.

### 4.7. Statistical Analysis and Data Presentation

Statistical analysis was performed with R software from the R Foundation for Statistical Computing (Vienna, Austria). Data were analyzed by one-way ANOVA. *p*-values ≤ 0.05 were considered statistically significant.

Figures show one out of at least three independent experiments providing similar results or the mean (±S.E.) of at least three experiments.

## 5. Conclusions

Inositols play well-established roles in fat metabolism, and their contribution has been extensively studied and described over the past fifty years [55], but our studies are the first to associate it with WAT/BAT trans-differentiation mediating by ERs. In fact, our results demonstrate that inositols could participate in WAT/BAT trans-differentiation through two different nuclear receptors: ERs and PPAR-γ. Moreover, both the inositol isomers tested seemed to participate in this process, even though to a different extent. These findings support the recommendation to further test both the isomers and/or their combination in preclinical studies to investigate whether these molecules can have positive effects in weight loss. Indeed, recent observations from Kulterer and coworker [56] demonstrated that BAT is less frequently detected in obese rather than in lean people. They also highlighted that BAT is associated with visceral adiposity rather than with total body fat content per se. Considering their findings and our results herein reported, we believe that our results open new avenues on the physiological role of inositol stereoisomers in energy metabolism. Moreover, although these in vitro results need further clinical verifications, our data offer numerous opportunities for the use of inositol stereoisomers in therapeutic strategies to help prevent or counteract obesity.

## Figures and Tables

**Table 1:** Primer sequences used in qPCR experiments (by primer-BLAST).

Gene	Protein Name	Sequence 5′→3′		Amplicon Length	NCBI Ref
ACTB	β-Actin	F: AGAAGGATTCCTATGTGGGGG	R: CATGTCGTCCCAGTTGGTGAC	101	NM_001101.5
ESR1	ER-α	F: GATGCTGAGCCCCCATACT	R: CACACGGCACAGTAGCGAG	128	NM_001122742.2
ESR2	ER-β	F: AGCTCAGCCTGTTCGA	R: TCTACGCATTTCCCCTCATCC	151	NM_001437.3
UCP-1	UCP-1	F: GGAAAGAAACAGCACCTAGTTT	R: CGTCAAGCCTTCGGTTGTTGCTA	197	NM_021833.5
PPARg-1	PPAR-γ1	F: CGTGGCCGCAGATTTGAA	R: CTTCCATTACGGAGAGATCCAC	166	NM_138712.5
PPARg-2	PPAR-γ2	F: GGTGAAACTCTGGGAGATTCT	R: CTCTGTGTCAACCATGGTCA	102	NM_015869.5

## Data Availability

Data are available from the corresponding author on a reasonable request.

## References

[1] Afshin A., Forouzanfar M.H., Reitsma M.B., Sur P., Estep K., Lee A., Marczak L., Mokdad A.H., Moradi-Lakeh M., Naghavi M. (2017). Health Effects of Overweight and Obesity in 195 Countries over 25 Years. N. Engl. J. Med..

[2] World Health Organization. https://www.who.int/.

[3] Nnodim J.O. (1987). Development of adipose tissues. Anat. Rec..

[4] Klaus S. (1997). Functional differentiation of white and brown adipocytes. Bioessays.

[5] Cannon B., Nedergaard J. (2004). Brown adipose tissue: Function and physiological significance. Physiol. Rev..

[6] Giralt M., Villarroya F. (2013). White, brown, beige/brite: Different adipose cells for different functions?. Endocrinology.

[7] Rosenwald M., Wolfrum C. (2014). The origin and definition of brite versus white and classical brown adipocytes. Adipocyte.

[8] Dragano N.R.V., Fernø J., Diéguez C., López M., Milbank E. (2020). Recent Updates on Obesity Treatments: Available Drugs and Future Directions. Neuroscience.

[9] Pak Y., Huang L.C., Lilley K.J., Larner J. (1992). In vivo conversion of [3H]myoinositol to [3H]chiroinositol in rat tissues. J. Biol. Chem..

[10] Facchinetti F., Appetecchia M., Aragona C., Bevilacqua A., Bezerra Espinola M.S., Bizzarri M., D’Anna R., Dewailly D., Diamanti-Kandarakis E., Hernández Marín I. (2020). Experts’ opinion on inositols in treating polycystic ovary syndrome and non-insulin dependent diabetes mellitus: A further help for human reproduction and beyond. Expert Opin. Drug Metab. Toxicol..

[11] Dinicola S., Unfer V., Facchinetti F., Soulage C.O., Greene N.D., Bizzarri M., Laganà A.S., Chan S.Y., Bevilacqua A., Pkhaladze L. (2021). Inositols: From Established Knowledge to Novel Approaches. Int. J. Mol. Sci..

[12] Milewska E.M., Czyzyk A., Meczekalski B., Genazzani A.D. (2016). Inositol and human reproduction. From cellular metabolism to clinical use. Gynecol. Endocrinol..

[13] Facchinetti F., Espinola M.S.B., Dewailly D., Ozay A.C., Prapas N., Vazquez-Levin M., Wdowiak A., Unfer V. (2020). Breakthroughs in the Use of Inositols for Assisted Reproductive Treatment (ART). Trends Endocrinol. Metab..

[14] Hayashi E., Maeda T., Tomita T. (1974). The effect of myo-inositol deficiency on lipid metabolism in rats. I. The alteration of lipid metabolism in myo-inositol deficient rats. Biochim. Biophys. Acta.

[15] Lee B.-J., Lee K.-J., Lim S.-J., Lee S.-M. (2008). Dietary myo-inositol requirement for Olive flounder, Paralichthys olivaceus (Temminch et Schlegel). Aquac. Res..

[16] Khosravi S., Lim S.-J., Rahimnejad S., Kim S.-S., Lee B.-J., Kim K.-W., Han H.-S., Lee K.-J. (2015). Dietary myo-inositol requirement of parrot fish, *Oplegnathus fasciatus*. Aquaculture.

[17] Fan C., Zhang D., Zhang J., Li J., Wang Y., Gao L., Han S. (2022). The effect of D-chiro-inositol on renal protection in diabetic mice. Aging.

[18] Montt-Guevara M.M., Finiguerra M., Marzi I., Fidecicchi T., Ferrari A., Genazzani A.D., Simoncini T. (2021). D-Chiro-Inositol Regulates Insulin Signaling in Human Adipocytes. Front. Endocrinol..

[19] Wabitsch M., Brüderlein S., Melzner I., Braun M., Mechtersheimer G., Möller P. (2000). LiSa-2, a novel human liposarcoma cell line with a high capacity for terminal adipose differentiation. Int. J. Cancer.

[20] Nicholls D.G., Locke R.M. (1984). Thermogenic mechanisms in brown fat. Physiol. Rev..

[21] Nedergaard J., Golozoubova V., Matthias A., Asadi A., Jacobsson A., Cannon B. (2001). UCP1: The only protein able to mediate adaptive non-shivering thermogenesis and metabolic inefficiency. Biochim. Biophys. Acta.

[22] Liu X., Zheng Z., Zhu X., Meng M., Li L., Shen Y., Chi Q., Wang D., Zhang Z., Li C. (2013). Brown adipose tissue transplantation improves whole-body energy metabolism. Cell Res..

[23] Wei G., Sun H., Liu J.L., Dong K., Liu J., Zhang M. (2020). Indirubin, a small molecular deriving from connectivity map (CMAP) screening, ameliorates obesity-induced metabolic dysfunction by enhancing brown adipose thermogenesis and white adipose browning. Nutr. Metab..

[24] Vijgen G.H., Bouvy N.D., Teule G.J., Brans B., Schrauwen P., van Marken Lichtenbelt W.D. (2011). Brown adipose tissue in morbidly obese subjects. PLoS ONE.

[25] Al-Qahtani S.M., Bryzgalova G., Valladolid-Acebes I., Korach-André M., Dahlman-Wright K., Efendić S., Berggren P.O., Portwood N. (2017). 17β-Estradiol suppresses visceral adipogenesis and activates brown adipose tissue-specific gene expression. Horm. Mol. Biol. Clin. Investig..

[26] Santos R.S., Frank A.P., Fátima L.A., Palmer B.F., Öz O.K., Clegg D.J. (2018). Activation of estrogen receptor alpha induces beiging of adipocytes. Mol. Metab..

[27] Nilsson M., Dahlman I., Rydén M., Nordström E.A., Gustafsson J.A., Arner P., Dahlman-Wright K. (2007). Oestrogen receptor alpha gene expression levels are reduced in obese compared to normal weight females. Int. J. Obes..

[28] Zhou Z., Moore T.M., Drew B.G., Ribas V., Wanagat J., Civelek M., Segawa M., Wolf D.M., Norheim F., Seldin M.M. (2020). Estrogen receptor α controls metabolism in white and brown adipocytes by regulating Polg1 and mitochondrial remodeling. Sci. Transl. Med..

[29] Rodríguez-Cuenca S., Monjo M., Gianotti M., Proenza A.M., Roca P. (2007). Expression of mitochondrial biogenesis-signaling factors in brown adipocytes is influenced specifically by 17beta-estradiol, testosterone, and progesterone. Am. J. Physiol. Endocrinol. Metab..

[30] Quarta C., Mazza R., Pasquali R., Pagotto U. (2012). Role of sex hormones in modulation of brown adipose tissue activity. J. Mol. Endocrinol..

[31] Yoshioka K., Yoshida T., Wakabayashi Y., Nishioka H., Kondo M. (1988). Reduced brown adipose tissue thermogenesis of obese rats after ovariectomy. Endocrinol. Jpn..

[32] Pedersen S.B., Bruun J.M., Kristensen K., Richelsen B. (2001). Regulation of UCP1, UCP2, and UCP3 mRNA expression in brown adipose tissue, white adipose tissue, and skeletal muscle in rats by estrogen. Biochem. Biophys. Res. Commun..

[33] Martínez de Morentin P.B., González-García I., Martins L., Lage R., Fernández-Mallo D., Martínez-Sánchez N., Ruíz-Pino F., Liu J., Morgan D.A., Pinilla L. (2014). Estradiol regulates brown adipose tissue thermogenesis via hypothalamic AMPK. Cell Metab..

[34] Zhang W., Schmull S., Du M., Liu J., Lu Z., Zhu H., Xue S., Lian F. (2016). Estrogen Receptor α and β in Mouse: Adipose-Derived Stem Cell Proliferation, Migration, and Brown Adipogenesis In Vitro. Cell. Physiol. Biochem..

[35] Monastra G., Vazquez-Levin M., Bezerra Espinola M.S., Bilotta G., Laganà A.S., Unfer V. (2021). D-chiro-inositol, an aromatase down-modulator, increases androgens and reduces estrogens in male volunteers: A pilot study. Basic Clin. Androl..

[36] Nordio M., Kumanov P., Chiefari A., Puliani G. (2021). D-Chiro-Inositol improves testosterone levels in older hypogonadal men with low-normal testosterone: A pilot study. Basic Clin. Androl..

[37] Gambioli R., Forte G., Aragona C., Bevilacqua A., Bizzarri M., Unfer V. (2021). The use of D-chiro-Inositol in clinical practice. Eur. Rev. Med. Pharmacol. Sci..

[38] Gambioli R., Montanino Oliva M., Nordio M., Chiefari A., Puliani G., Unfer V. (2021). New Insights into the Activities of D-Chiro-Inositol: A Narrative Review. Biomedicines.

[39] Montanino Oliva M., Gambioli R., Forte G., Porcaro G., Aragona C., Unfer V. (2022). Unopposed estrogens: Current and future perspectives. Eur. Rev. Med. Pharmacol. Sci..

[40] Semple R.K., Chatterjee V.K., O’Rahilly S. (2006). PPAR gamma and human metabolic disease. J. Clin. Investig..

[41] Gervois P., Torra I.P., Fruchart J.C., Staels B. (2000). Regulation of lipid and lipoprotein metabolism by PPAR activators. Clin. Chem. Lab. Med..

[42] Zhang Y., Li C., Zhang W., Zheng X., Chen X. (2020). Decreased Insulin Resistance by Myo-Inositol Is Associated with Suppressed Interleukin 6/Phospho-STAT3 Signaling in a Rat Polycystic Ovary Syndrome Model. J. Med. Food.

[43] Klusóczki Á., Veréb Z., Vámos A., Fischer-Posovszky P., Wabitsch M., Bacso Z., Fésüs L., Kristóf E. (2019). Differentiating SGBS adipocytes respond to PPARγ stimulation, irisin and BMP7 by functional browning and beige characteristics. Sci. Rep..

[44] Mukherjee R., Jow L., Croston G.E., Paterniti J.R. (1997). Identification, characterization, and tissue distribution of human peroxisome proliferator-activated receptor (PPAR) isoforms PPARgamma2 versus PPARgamma1 and activation with retinoid X receptor agonists and antagonists. J. Biol. Chem..

[45] Mueller E., Drori S., Aiyer A., Yie J., Sarraf P., Chen H., Hauser S., Rosen E.D., Ge K., Roeder R.G. (2002). Genetic analysis of adipogenesis through peroxisome proliferator-activated receptor gamma isoforms. J. Biol. Chem..

[46] Sabichi A.L., Subbarayan V., Llansa N., Lippman S.M., Menter D.G. (2004). Peroxisome proliferator-activated receptor-gamma suppresses cyclooxygenase-2 expression in human prostate cells. Cancer Epidemiol. Biomark. Prev..

[47] Tontonoz P., Hu E., Graves R.A., Budavari A.I., Spiegelman B.M. (1994). mPPAR gamma 2: Tissue-specific regulator of an adipocyte enhancer. Genes Dev..

[48] Fischer-Posovszky P., Newell F.S., Wabitsch M., Tornqvist H.E. (2008). Human SGBS cells—A unique tool for studies of human fat cell biology. Obes. Facts.

[49] Wabitsch M., Brenner R.E., Melzner I., Braun M., Möller P., Heinze E., Debatin K.M., Hauner H. (2001). Characterization of a human preadipocyte cell strain with high capacity for adipose differentiation. Int. J. Obes. Relat. Metab. Disord..

[50] Garzon S., Laganà A.S., Monastra G. (2019). Risk of reduced intestinal absorption of myo-inositol caused by D-chiro-inositol or by glucose transporter inhibitors. Expert. Opin. Drug Metab. Toxicol..

[51] Ranaldi G., Ferruzza S., Natella F., Unfer V., Sambuy Y., Monastra G. (2020). Enhancement of D-chiro-inositol transport across intestinal cells by alpha-Lactalbumin peptides. Eur. Rev. Med. Pharmacol. Sci..

[52] Monastra G., Sambuy Y., Ferruzza S., Ferrari D., Ranaldi G. (2018). Alpha-lactalbumin Effect on Myo-inositol Intestinal Absorption: In Vivo and In Vitro. Curr. Drug Deliv..

[53] Rousset S., Mozo J., Dujardin G., Emre Y., Masscheleyn S., Ricquier D., Cassard-Doulcier A.M. (2007). UCP2 is a mitochondrial transporter with an unusual very short half-life. FEBS Lett..

[54] Moazed B., Desautels M. (2002). Differentiation-dependent expression of cathepsin D and importance of lysosomal proteolysis in the degradation of UCP1 in brown adipocytes. Can. J. Physiol. Pharmacol..

[55] Hawthorne J.N. (1972). Inositol lipid metabolism and cell membrane. Biochem. J..

[56] Kulterer O.C., Herz C.T., Prager M., Schmöltzer C., Langer F.B., Prager G., Marculescu R., Kautzky-Willer A., Hacker M., Haug A.R. (2022). Brown Adipose Tissue Prevalence Is Lower in Obesity but Its Metabolic Activity Is Intact. Front. Endocrinol..

